# Positive Outcomes Influence the Rate and Time to Publication, but Not the Impact Factor of Publications of Clinical Trial Results

**DOI:** 10.1371/journal.pone.0054583

**Published:** 2013-01-30

**Authors:** Pilar Suñé, Josep Maria Suñé, J. Bruno Montoro

**Affiliations:** 1 Pharmacy Department, Hospital Universitari Vall d’Hebron, Barcelona, Spain; 2 Pharmacy Faculty, Universitat de Barcelona, Barcelona, Spain; Johns Hopkins Bloomberg School of Public Health, United States of America

## Abstract

**Objectives:**

Publication bias may affect the validity of evidence based medical decisions. The aim of this study is to assess whether research outcomes affect the dissemination of clinical trial findings, in terms of rate, time to publication, and impact factor of journal publications.

**Methods and Findings:**

All drug-evaluating clinical trials submitted to and approved by a general hospital ethics committee between 1997 and 2004 were prospectively followed to analyze their fate and publication. Published articles were identified by searching Pubmed and other electronic databases. Clinical study final reports submitted to the ethics committee, final reports synopses available online and meeting abstracts were also considered as sources of study results. Study outcomes were classified as positive (when statistical significance favoring experimental drug was achieved), negative (when no statistical significance was achieved or it favored control drug) and descriptive (for non-controlled studies). Time to publication was defined as time from study closure to publication. A survival analysis was performed using a Cox regression model to analyze time to publication. Journal impact factors of identified publications were recorded. Publication rate was 48·4% (380/785). Study results were identified for 68·9% of all completed clinical trials (541/785). Publication rate was 84·9% (180/212) for studies with results classified as positive and 68·9% (128/186) for studies with results classified as negative (p<0·001). Median time to publication was 2·09 years (IC95 1·61–2·56) for studies with results classified as positive and 3·21 years (IC95 2·69–3·70) for studies with results classified as negative (hazard ratio 1·99 (IC95 1·55–2·55). No differences were found in publication impact factor between positive (median 6·308, interquartile range: 3·141–28·409) and negative result studies (median 8·266, interquartile range: 4·135–17·157).

**Conclusions:**

Clinical trials with positive outcomes have significantly higher rates and shorter times to publication than those with negative results. However, no differences have been found in terms of impact factor.

## Background

Evidence-based medical decisions rely on the absence of bias in the available published results. When the publication or not of a particular study is determined by its outcomes or statistical significance, the derived bias is unacceptable, since it results in an exaggeration of the positive effects of a given treatment [1], [2].

Relevance of publication bias, in any of its manifestations, is undoubtedly more serious when it relates to clinical trials involving treatments in more advance phases of development, newly approved drugs, or new indications for registered drugs, since it may directly result in the use of less cost-effective, ineffective, or even harmful interventions in clinical practice [1].

Efforts have been made to prevent and reduce publication bias, including prospective registration of clinical trials [3], [4], publication of results in public databases [5] or in sponsor websites [6], and journal editors’ initiatives to promote trial registration [7]. However, a single universal tool to provide access to all clinical trial results, regardless of their outcomes, is not currently available.

Previous research has documented the existence of lower publication rates [8]–[12] and delay of publication [9], [13] in studies with negative outcomes in comparison with studies with positive outcomes. However, to our knowledge, no previous studies have assessed publication bias in a large cohort of drug-related clinical trials with known results, both positive and negative.

Direction and strength of outcomes might also affect the availability and extent of dissemination of study results as measured by article impact factor. Few studies have assessed the effect of study outcomes on impact factors, showing a tendency to publish studies with positive results in high impact factor journals [14], [15].

The aim of this study was to assess whether study outcomes affect the dissemination of clinical trials findings, in terms of rate, time to publication, and journal impact factor, in a cohort of clinical trials with known results, publically available or not. Furthermore, to evaluate the extent of publication bias in trials with a greater relevance on clinical practice, two subpopulation analyses were performed, with phase 3 and 4 clinical trials, and with trials in which conditions of use of experimental drugs had been approved, before or after trial completion.

## Methods

### Source Data

All drug-evaluating clinical trials submitted to and approved by the Ethics Committee (EC) of Hospital Vall d’Hebron in Barcelona, Spain, between 1997 and 2004 were prospectively followed to analyze their fate, results if available, and subsequent publication. The following information was recorded: experimental drug name, medical condition, name and specialty of principal investigator, main outcome, sample size, type of sponsor (industry or no industry), phase of study, study design and dates of EC approval, trial initiation and closure. Conditions of use of experimental drugs were classified as approved or off label, according to label information at the time of trial submission. Current status of the trial was recorded at the time of database closure (February 2010), categorized as not initiated, completed, prematurely terminated, or ongoing. Final reports submitted to the EC by the sponsor were also prospectively collected and filed for ulterior analysis of results.

### Study Results Search Strategy

Completed and prematurely terminated studies were classified as published when at least one article was identified in peer reviewed literature. Other sources of results, if found, were also recorded: meeting abstracts, online final report synopses, and final reports submitted by the sponsor to the EC.

We searched clinicaltrials.gov site to identify registered trials and study publications, if mentioned, using drug name, medical condition, sponsor identification and protocol number as keywords. Additionally, literature searches were performed to identify trial publications in the following databases of biomedical journals: Pubmed, ISI web of knowledge and Cochrane Library Plus. Keywords used in the search were drug name, medical condition, sponsor name, principal investigator, and clinicaltrials.gov identification number, when available. If no hits were found in the first strategy, a second search was performed, using only drug name and medical condition as keywords. We also reviewed Spanish language databases, Indice Médico Español and the Spanish Ministry of Education doctoral thesis database (TESEO) for additional publications.

Criteria used to match identified publications to trials were classified as fixed, which should be included in the publication according to CONSORT guidelines [16], and optional, when their inclusion in the article was per author decision. Fixed criteria included identification of tested drugs (dosage and schedules), study population (inclusion criteria and medical condition), sample size, main outcome, statistical analysis and sponsor or funding source. Optional criteria were protocol number or clinicaltrials.gov identification, and presence of the hospital and principal investigator name in the list of participants.

We also checked sponsor websites and the online database www.clinicalstudyresults.org to locate study final report synopses, using drug name, medical condition, protocol number, and clinicaltrials.gov identification number, when available, as keywords.

Last search was performed during March 2010.

The following information was collected for identified publications: date of publication, journal name, and journal impact factor, according to the 2008 list of journal impact factors [17].

### Classification of Study Outcomes

For each trial, identified result sources were recorded and quantified.

Results were classified as: 1) complete, 2) partial, when analysis of results was preliminary or incomplete, or 3) not available, when although the source of results was identified, it was not possible to access the full text information.

For classification of results, we searched for significance on the main outcome as defined in the original study protocol. When no clear single main outcome was defined on the protocol, i.e. multiple variables or multi-arm studies, the trial was considered positive if any major outcome reached statistical significance favoring the experimental group. When the only source of study results was meeting abstract or journal publication, and the main outcome was different than that defined on the protocol, the outcome chosen to classify study results was that defined as main outcome in the publication.

Study main outcomes were thus classified: as 1) positive (when statistical significance favoring experimental drug was achieved, p<0·05), 2) negative (when no statistical significance was achieved or it favored control drug), and 3) descriptive (for non-controlled studies). For trials designed as equivalence or noninferiority studies, results were classified as positive when no significant differences were found between the comparison treatment groups, and as negative when they failed to prove the equivalence or noninferiority criteria as defined by the study design.

### Post-trial Drug Registration

Labels of experimental drugs were searched in the European Medicine Agency (EMA) database in order to check whether conditions of use had been approved in the European Union upon trial completion. Last search was performed in March 2010.

### Statistical Analysis

Descriptive analysis was performed for all variables. For continuous variables, mean, median interquartile range, standard deviation, and confidence intervals were calculated. Tables of frequencies were elaborated for categorical variables.

Publication rates were compared with the use of the chi-square test and Fisher’s exact test.

The Kaplan Meier method was used to analyze time to publication. The influence of outcomes –positive, negative, and descriptive- was evaluated with the use of the log-rank test. Hazard ratios and confidence intervals were calculated on the basis of a Cox regression model. Trial characteristics included in the analysis were sponsorship type (industry or non-industry), phase of development (1/2 or 3/4), sample size (categorized in four groups: less than 100 subjects, 100 to 500 subjects, 500 to 1000 subjects, and more than 1000 subjects), and medical specialty.

Differences on impact factor were studied through nonparametric tests (U Mann Whitney or Kruskal Wallis) since the variable did not follow normal distribution.

Primary analysis or rate and time to publication was performed with the total population of completed trials. Secondary analyses were also performed with the phase 3/4 completed trial subpopulation and with the population of completed trials with drugs whose conditions of use were already approved or had been approved by regulatory authorities after trial completion.

Primary analysis of impact factor was performed with the total population of published trials, and secondary analysis were performed with phase 3/4 published trials and published trials whose conditions of use were already approved or had been approved by regulatory authorities after trials completion.

Software used for statistical analysis was SPSS version 16.0 (SPSS Inc, Chicago, IL).

## Results

**Figure 1:**
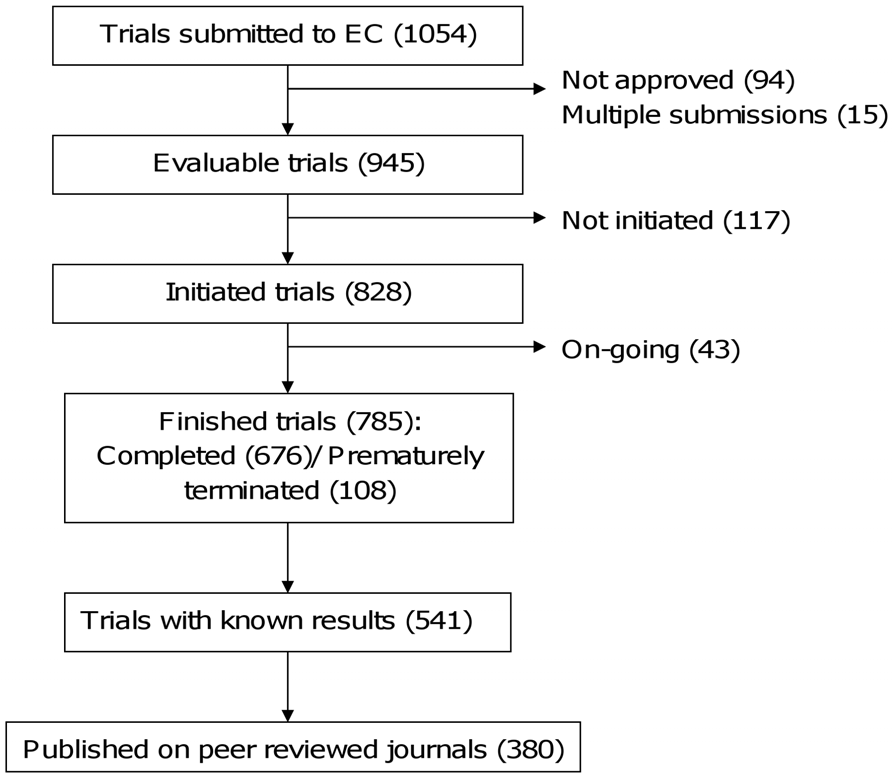
Flowchart of evaluated clinical trials.

During the study, 1054 drug-related clinical trials were submitted to the Ethics Committee (EC). Ninety four were not approved by the EC and 15 corresponded to duplicate submissions. The remaining 945 clinical trials were included in the analysis. Of these, 117 were never initiated and 43 were still ongoing by the end of February 2010. Of the remaining 785 studies, 677 were completed according to protocol, while 108 were prematurely terminated (figure 1). Two hundred and fifty nine of all 945 evaluated trials (27·4%) were registered on clinicaltrials.gov.

### Descriptive Analysis

Characteristics of the 785 finished studies, those with known results and published trials are recorded on Table 1. Completed trials were mostly industry sponsored (89%), controlled (75%), phase 3/4 (76%), with a recruitment between 100 to 500 subjects (42%), and evaluating unregistered drugs (62%). Overall, 41 different medical specialties were involved, being the most prevalent, oncology (160), neurology (90), infectious diseases (59), and cardiology (55). A total of 431 different chemical entities were evaluated as experimental treatment. Median duration of all completed clinical trials was 2·03 years (range 115 days–11·3 years).

**Table 1:** Characteristics of finished studies, studies with known results and published studies.

	Finished studies (n = 785)	Studies with known results (n = 537)^2^	Published studies (n = 380)
Prematurely terminated	108 (13·8%)	50 (9·3%)	23 (6·1%)
Type of sponsorship			
Industry	697 (88·8%)	487 (90·7%)	341 (89·7%)
Non-industry	88 (11·2%)	50 (9·3%)	39 (10·3%)
Phase of study			
1/2	184 (23·4%)	126 (23·5%)	76 (20·0%)
3/4	601 (76·6%)	411 (76·5%)	304 (80·0%)
Study design			
Controlled	590 (75·2%)	417 (77·7%)	315 (82·9%)
Uncontrolled	195 (24·8%)	120 (22·3%)	65 (17·1%)
Sample size^1^			
Less than 100	187 (23·8%)	115 (21·4%)	72 (18·9%)
100 to 500	333 (42·4%)	228 (42·5%)	155 (40·8%)
500 to 1000	146 (18·6%)	106 (19·7%)	78 (20·5%)
More than 1000	100 (12·7%)	87 (16·2%)	75 (19·3%)
Conditions of use of study drug			
Registered drugs. Approved use	79 (10·1%)	49 (9·1%)	34 (8·9%)
Registered drugs. Off label use	222 (28·3%)	162 (30·2%)	117 (30·8%)
Unregistered drugs	481 (61·7%)	326 (60·7%)	229 (60·3%)

1 Trial sample size unknown for 19 studies.

2 4 trials with results located but unavailable for analysis.

**Figure 2:**
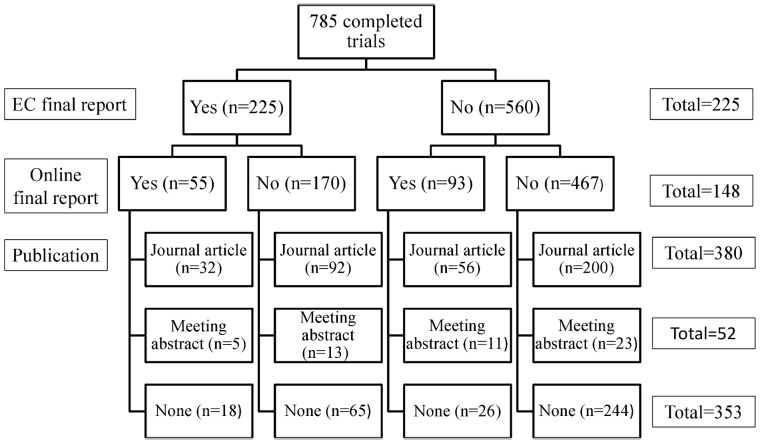
Flowchart of study results research.

Results were identified for 541 of the 785 completed trials. The most frequent source of results were peer reviewed journals (380), followed by final reports submitted to EC (225), online clinical study results synopsis (148), and meeting abstracts (52) (figure 2). For 314 trials only one source of results was located (65 study final reports, 200 journal articles, 26 online final reports and 23 congress abstracts). One hundred and ninety had two sources of results and for 37 completed trials we found three different sources of results. Full text of results could not be accessed for 4/541 studies and 22/541 had null or partial analyses. Of these 22 trials, 9 corresponded to prematurely terminated studies, while for the other 13 only preliminary reports could be identified. Analysis of the 515 studies with full text results and complete analyses resulted in 212 studies with positive results, 186 with negative results, and 117 with descriptive results.

Seventy nine of the 785 completed trials evaluated drugs in indications already approved, while 222 evaluated registered drugs in unapproved indications, and 484 investigated previously unregistered investigational drugs. Analysis of experimental drug labels upon completion of the trial revealed that for 245 of all completed trials evaluating off label drug uses and unregistered drugs, the investigated drug use had been approved by regulatory authorities (34·7%).

### Publication Rate

Publication rate in peer reviewed journals was 48·5% (380/785) for all completed trials, and 21·3% for prematurely terminated studies (23/108). In the phase 3 or 4 trials subgroup, publication rate was 50·6% (304/601), and 56·8% (184/324) for trials with drugs whose conditions of use were already approved or had been approved by regulatory authorities after trial completion.

Publication rate was 84·9% for studies with positive results (180/212) and 68·8% (128/186) for studies with negative results. Sixty nine of the 117 studies with descriptive results were published (59%) (Table 2). Trials with positive results were more likely to be published than those with negative results, in the total population of finished trials (OR = 2·55; IC95∶ 1·56–4·15), in the phase 3 or 4 trial subpopulation (OR = 2·80; IC95∶ 1·62–4·84), and in the population of trials evaluating subsequently approved drugs and indications (OR = 4·28; IC95∶ 1·84–10·00).

**Table 2:** Publication rates of completed trials with known results.

	All trials (n = 515)		Phase 3 and 4 trials(n = 402)		Trials with approved drugs and uses (n = 233)	
	Total	Published	Total	Published	Total	Published
Positive results	212	180 (84·9%)	190	165 (86·8%)	132	120 (90·9%)
Negative results	186	128 (68·8%)	151	106 (70·2%)	50	35 (70·0%)
Results	117	69 (59·0%)	61	31 (50·8%)	51	27 (52·9%)

26 trials with null analysis or results not available were excluded (including 3 published studies).

### Time to Publication

Mean follow-up time of trials since their completion was 4·24 years (IC95∶ 4·03–4·46) with a range of 8 days to 12·19 years.

**Figure 3:**
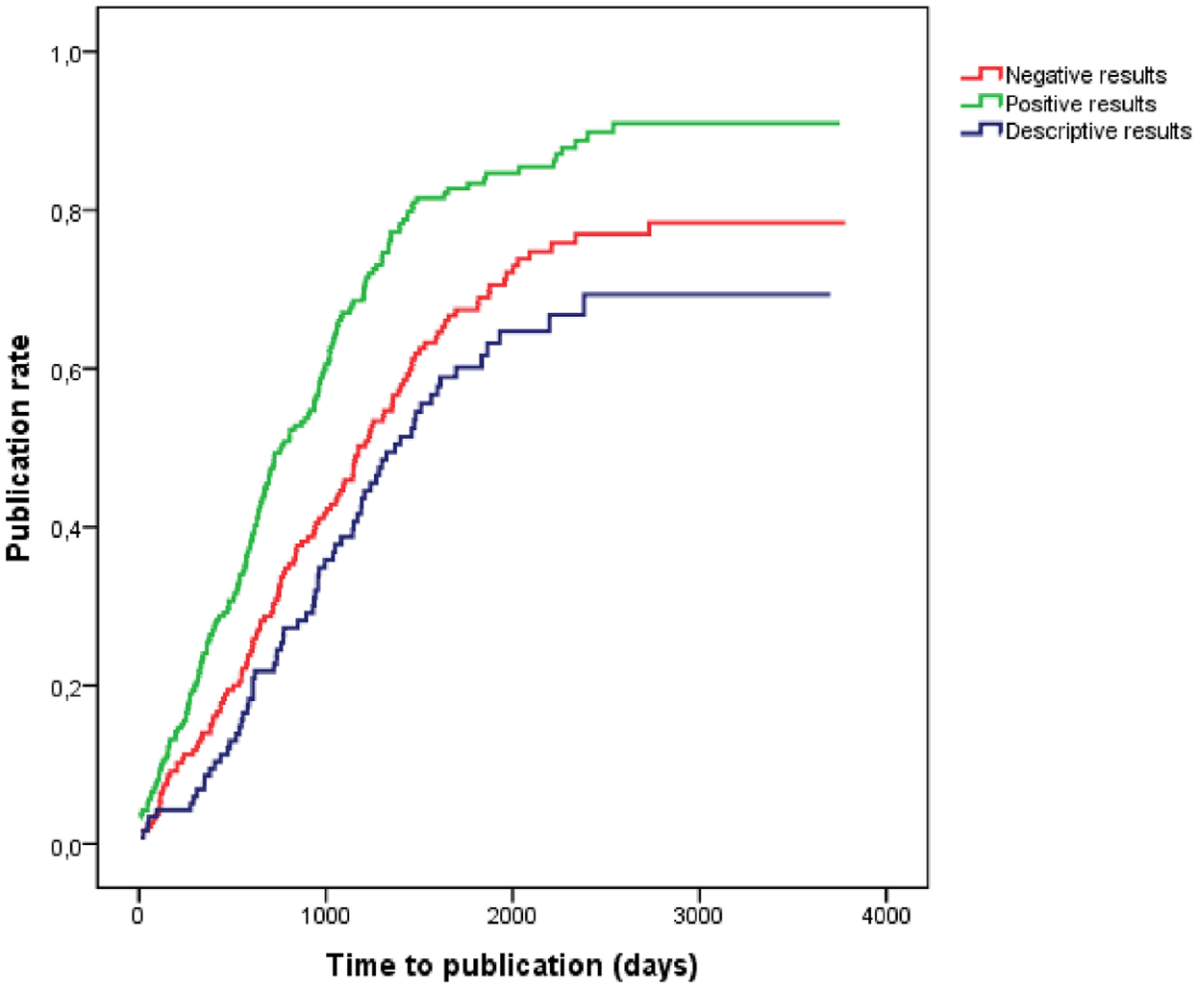
Kaplan Meier survival curves of time to publication sorted by study outcomes (positive, negative, descriptive).

Median survival time (MST) from study completion to publication was 1081 days (2·96 years) (IC95∶ 2·68–3·24) for all trials. Positive trials were published significantly earlier (MST = 2·09 years; IC95∶ 1·61–2·56) than negative (MST = 3·21 years; IC95∶ 2·69–3·74), and descriptive (MST = 3·75 years; IC95∶ 3·04–4·46) (p<0·001) (Figure 3).

After adjusting per sponsorship type, phase of study, sample size and medical specialty, an association between study outcomes and publication time exists, with a hazard ratio of 1·99 (IC95∶ 1·55–2·55) favouring positive over negative studies (Table 3). These results were consistent on the subsequent sub-analyses performed on phase 3 and 4 trials (HR = 2·11; IC95∶ 1·61–2·77), and trials involving approved indications and drugs (HR = 2·43; IC95∶ 1·56–3·79).

**Table 3:** Predictors of time to publication.

	All trials (n = 785)		Phase 3 and 4 trials (n = 601)		Trials with approved drugs and uses (n = 324)	
	HR (IC95)	P	HR (IC95)	p	HR (IC95)	p
Negative outcome	1		1		1	
Positive outcome	1·99 (1·55–2·55)	<0·001	2·11 (1·61–2·77 )	<0·001	2·43 (1·56–3·79)	<0·001
Descriptive outcome	0·90 (0·64–1·27)	0·57	0·75 (0·49–1·15 )	0·19	0·78 (0·44–1·37)	0·38
Industry-sponsored	1		1		1	
Nonindustry-sponsored	1·93 (1·33–2·80)	0·001	1·90 (1·28–2·83)	0·001	1·18 (0·63–2·20)	0·61
Sample size <100	1		1		1	
Sample size 100 to 500	0·89 (0·64–1·24)	0·50	0·81 (0·54–1·21 )	0·30	0·90 (0·48–1·69)	0·74
Sample size 500 to 1000	0·98 (0·67–1·43)	0·93	0·90 (0·58–1·39)	0·63	1·12 (0·56–2·25)	0·74
Sample size >1000	2·52 (1·67–3·81)	<0·001	2·37 (1·49–3·77)	<0·001	2·45 (1·18–5·10)	0·02
Phase 1/2	1		–	–	1	
Phase 3/4	1·00 (0·74–1·36)	0·98	–	–	0·71 (0·38–1·31)	0·27

Non industry sponsorship, and a sample size larger than 1000 subjects were also associated with shorter publication times (HR = 1·93; IC95∶ 1·33–2·80 and HR = 2·52; IC95∶ 1·67–3·81, respectively). The association of a larger sample size and time to publication was consistent in all three analyses, while the sponsorship association was not present in the approved drug trials subgroup.

### Impact Factor

The 380 published trials were published in 125 different journals. Journals with the highest number of published trials were New England Journal of Medicine (47), Journal of Clinical Oncology (38), The Lancet (23) and Annals of Oncology (12).

Median (interquartile range[IQR]) impact factor (IF) for all 380 published articles was 6·448 (3·568–17·157). Comparison of IF values according to study outcomes revealed no differences in between positive (median: 6·308, IQR: 3·141–28·409) and negative trials (median: 8·266, IQR: 4·135–17·157), while for descriptive trials mean IF was significantly lower (median: 4·935, IQR: 3·498–10·313).

In phase 3 and 4 trials, higher IF values were found for trials with negative results, with statistically significant differences between all groups, while in the subgroup of trials with approved drugs, no differences were found (Table 4).

**Table 4:** Impact factor of publications.

Study results	Median (IQR)	p
All trials		
Positive (n = 180)	6·308 (3·141–28·409)	0·173(1)
Negative (n = 128)	8·266 (4·135–17·157)	
Descriptive (n = 68)	4·935 (3·498–10·313)	0·012(2)
Global (n = 376)	6·448 (3·568–17·157)	
Phase 3 or 4 trials		
Positive (n = 165)	6·030 (3·082–22·933)	0·027(1)
Negative (n = 106)	9·863 (4·267–28·409)	
Descriptive (n = 30)	4·206 (2·912–5·252)	<0·001(2)
Global (n = 303)	6·325 (3·164–17·157)	
Trials with approved drugs and uses		
Positive (n = 120)	5·839 (3·064–17·457)	0·896(1)
Negative (n = 35)	7·056 (2·704–17·157)	
Descriptive (n = 27)	4·935 (2·970–17·157)	0·537(2)
Global (n = 184)	5·613 (2·979–17·157)	

3 trials with null/partial/non available analysis and 1 trial published in a journal not listed on 2008 impact factor index were excluded.

(1) Comparison between studies with positive and negative results.

(2) Comparison between studies with positive, negative and descriptive results.

## Discussion

The results of this long-term, extensive study, evidence that available published clinical trial results do not match the originally generated outcomes, since negative results are published significantly less and later than positive results, compromising evidence based medical decisions. Interestingly, no differences have been found on impact factor of publications of clinical trials with positive or negative results, which seems to indicate that overall, statistical significance of results is not a major reason for rejection of clinical trial articles by journal editors.

Clinical trial publication is not a clearly dichotomic process [1], [18]. Results may appear in a number of different forms of presentation, which include, but are not restricted to medical journal articles. In order to assess publication bias, we considered different sources of presentation of results, which include final clinical study reports addressed to the EC, synopsis of results made available online by the sponsors, meeting abstracts, and especially, journal articles. However, a study has been classified as published only when global results of the trial have appeared as an article in a medical journal, since it has been considered that only original articles contain public information sufficiently detailed to allow decision making [19]. It should be noted that journal articles, in contrast to data submitted online by sponsoring companies or available as meeting abstracts, have a previous review process that guarantees quality and completeness of the information provided. A documented phenomenon [20]–[23] not foreseen in the original design of this study was that comparison between publications and original protocols revealed in some cases differences in main outcomes presented in the journal article. Quantifying and analyzing this phenomenon was beyond the scope of this study, and in those cases, a decision was made to prioritize the main outcome as presented in the article in order to classify study results. Thus, in some cases, a trial classified as positive might have been negative if the protocol main outcome had been taken into consideration.

Out of the 785 completed clinical trials, the results of 244 (31%) could not be identified by any of the means of results presentation investigated in this study. Fifty eight of these 244 were prematurely cancelled, but the other 186 were completed according to protocol, which means there is a substantial amount of information missing regarding clinical trials that were performed and completed. A possibility of mismatching and even misidentification of publication exists [20]–[25], although we believe we have partially overcome this problem by using multiple criteria to match publications with protocols. Another potential weakness of this study comes from the censoring date used. The follow up time since study submission was decided in order to allow for the maximum number of studies to be completed and published. Since we are aware that these data might change overtime, a survival study design was applied to analyze this data, in order to add validity to our results. It should also be noted that investigator – and more importantly- sponsor reasons for not dissemination of study results – other than the direction of results- were not investigated in this study.

Previous studies evidencing publication bias have been very diverse in terms of research questions, design and study characteristics, and thus have limitations in terms of validity. The present study has used a single wide cohort of clinical trials which were followed since their inception, and has quantified publication bias through the fusion of results published on medical journals and results obtained through other routes, including a non-public cohort, less susceptible to biases, constituted by clinical final reports submitted to the EC.

Our study provides clear evidence of the existence of publication bias favoring positive result studies over negative. Previous studies, performed in different areas of clinical research, including basic experimental studies, observational studies, and clinical trials, have shown the existence of a positive association between publication rates and favorable outcomes [8]–[12]. Publication bias may be quantified not only through publication rates, but also through the speed with which results are made available. When time of publication depends on the nature of results, this phenomenon is qualified as time lag bias [26], [27]. In our study, mean survival time to publication has been over one year shorter for positive studies than for negative studies. Previous studies, using smaller cohorts of clinical trials, have confirmed an association between study results over time to publication [9], [13]. However, the findings of these studies cannot be compared with our results, due to the different criteria used to measure time to publication. Given the high variability in study duration, we considered more appropriate to measure time to publication as the interval between date of study closure (end of follow-up) and date of publication.

Studies with a descriptive hypothesis have shown lower publication rates and longer times to publication, which is indicative of a lesser priority applied to this type of studies in comparison to those with a comparative hypothesis. However, surprisingly, extent of publication and time lag bias has been greater when analyzing only phase 3/4 trials and those whose conditions of use have been approved after the trial, since this is a population of trials with a major influence on clinical practice. These results confirm the findings of a previous study evaluating the rate of publication of 909 trials supporting new drugs approved by the FDA and in which it was reported that rate of publication was associated with significance of outcomes [28]. Undoubtedly, the application of the FDA Amendments Act of 2007, which mandates basic public results reporting for all trials supporting FDA-approved drugs, will have an effect on this scenario in the future. As other authors have pointed out [28], this information will be publically available, but it is unknown whether -or how/when- negative trials data will translate into journal article publications.

Impact factor is an index based on the frequency with which a journal article is cited on scientific publication, and is considered a marker of journal quality [29]. There is a possibility of bias during review process previous to publication, if the reasons for rejection or acceptation are related to study results, independently of the scientific quality of the study. Four studies that examined manuscripts submitted to different journals concluded that manuscript acceptation was not associated with the statistical significance of the studies [30]–[33]. However, experimental cohort studies on published trials seem to indicate otherwise [3], [14], [15].

Our study has found no differences in impact factor of publication of clinical trials with positive or negative results, while for descriptive trials the values of impact factor have been significantly lower. Moreover, in phase 3 and 4 trials, higher impact factor values were found for trials with negative results. This would seem to indicate that publication bias is set, not at the moment of selection of articles by journal editors, but previously, when the decision to submit or not manuscripts for publication is taken, as other authors have stated [12], [34].

The design of a study of these characteristics, involving follow-up of trials since their inception, requires a considerable amount of time of execution, to allow for a sufficient time in order for the trials to be completed and published, as is the case for this cohort of studies, which started in 1997. Further research is needed to evaluate the effect of the newly initiatives destined to increase study results transparency, such as prospective registration of clinical trials, open access to results policy, and improved trial publication guidelines.

The results of this study point out to the fact that a change in paradigm is needed when access to clinical trial results is concerned. All actors implicated, investigators, regulatory authorities, journal editors, and especially, sponsoring companies, should provide for means to guarantee and increase public availability of unpublished results.
